# Prediction and therapeutic targeting of the tumor microenvironment-associated gene CTSK in gastric cancer

**DOI:** 10.1007/s12672-023-00821-0

**Published:** 2023-11-06

**Authors:** Zilong Bai, Chunyu Yan, Dongmin Chang

**Affiliations:** 1 Department of Surgical Oncology, The First Affiliated Hospital of Xi’an Jiaotong University, Xi’an, 710061 Shanxi China; 2 Department of Geriatric Endocrinology, The First Affiliated Hospital of Xi’an Jiaotong University, Xi’an, 710061 Shanxi China

**Keywords:** Gastric cancer, CTSK, Immune infiltration, Tumor microenvironment, Biomarker

## Abstract

**Background:**

Cathepsin-K (CTSK) is overexpressed in Gastric cancer (GC) and the mechanism of its overexpression in GC is still unclear. The present work found CTSK as a potential predictive biomarker and immunotherapeutic target for GC based on the tumor microenvironment (TME).

**Methods:**

From public databases, gene expression profiles and clinical data of GC were downloaded to analyze the distribution of stromal and immune cells and tumor abundance in TME. Differentially expressed genes (DEGs) associated with TME were obtained by differential analysis, followed by cross-screening to obtain CTSK as a gene associated with TME. Next, a series of methods and tools were employed to explore the relationships between clinicopathological features of GC and CTSK expression as well as prognosis, tumor immune microenvironment, immune checkpoints and drug sensitivity. And GSEA was used to investigate the potential role of CTSK in the tumor microenvironment of GC.

**Results:**

From the dataset, we obtained a total of 656 DEGs associated with TME and the stromal component of TME was found to be closely involved in GC prognosis. CTSK was cross-screened as the key gene associated with TME by the PPI network and univariate Cox regression analysis. Pan-cancer analysis revealed significant high expression of CTSK in a variety of cancers. Subsequently, we hypothesized that high-expressed CTSK was closely correlated with poor prognosis and lymph node metastasis of tumors, and that CTSK, a GC TME-related gene, was largely involved in a range of biological behaviors of tumors, with a significant correlation between several immune cells.

**Conclusion:**

CTSK was validated as a potential prognostic biomarker related to TME of GC and could be a promising next-generation immunotherapeutic target for GC.

**Supplementary Information:**

The online version contains supplementary material available at 10.1007/s12672-023-00821-0.

## Introduction

Gastric cancer (GC) ranked the fifth most frequently detected cancer and the third largest cause of cancer morality all over the world [1]. GC is also a leading cause of cancer deaths in Chinese society [2]. Although the incidence of GC in China is experiencing a decline, most GC patients are still diagnosed when they are at an advanced stage, facing limited treatment options and a poor prognosis [3]. With timely radical surgery after diagnosis, the 5-year survival rate can exceed 90% for early-stage GC patients [4], however, most patients are already past the early stage when detected because of the insidious onset and rapid progression of cancer [5]. For patients with advanced GC, the median overall survival rate is approximately 8 months [6]. As first-line treatment for patients with advanced cancer, current use of targeted therapies could improve clinical outcomes, but its screening of the beneficiary population and propensity for drug resistance has effectively improved the prognosis of patients [5]. Thus, we need to explore and develop new early diagnosis and prognostic prediction strategies for GC in order to improve the survival time as well as patients’ quality of life of patients.

TME is known to be the environment in which tumor cells or tumor stem cells grow, and is therefore also closely related to metastasis, tumorigenesis, and progression. Evidence increasingly indicates the key role of TME in cancer progression and treatment [7, 8]. TME is complex in its composition and includes both cancer cells and stromal cells such as cancer-associated macrophages, fibroblasts, as well as infiltrating immune cells, neovascularization and extracellular matrix [9]. Direct or indirect interactions between cancer cells and components in TME can modulate a variety of biological behaviors in tumors, such as induction of proliferation and inhibition of apoptosis, promotion of distant metastasis and induction of immune tolerance [10]. In addition, the two main components of TME, immune cells and stromal cells, are the key components in the diagnosis and prognosis of tumors and have a profound impact on tumorigenesis, progression and treatment resistance [11, 12]. Current research suggests that stromal cells can be involved in tumor invasion and spread via the secretion of a range of factors, for example, growth factors, chemokines and cytokines [13].Tumor tissue has been found by some studies to be associated with immune cell infiltration in TME, which will largely affect clinical cancer treatment outcome as well as survival prognosis [14–16]. CXCR4 may be a potent tumor and prognostic marker for TME, not only involved in the biological behavior associated with GC but also closely associated with immune cells in TME [17]. This indicates that a significant proportion of TME-related tumor markers play an essential role in GC. Therefore, targeting key molecules in TME and inhibiting their interactions may be a new therapeutic strategy.

This study used GC patients’ gene expression data provided by public database to determine tumor purity through the calculation of the stromal and immune cell profiles in tumor tissues using the ESTIMATE algorithm and explored the association between the stromal and immune components of TME and the clinicopathological features and prognosis of GC. Differential genes were then screened in terms of stromal and immune scores, and key biological processes and pathways of DEGs were analyzed by functional enrichment analysis. In addition, CTSK, the only gene associated with TME, was extracted by combined screening of DEGs by COX regression analysis and development of a PPI network. The potential link between immune cells and CTSK expression was next assessed by the CIBERSORT algorithm. We hypothesized that CTSK may be a potential prognostic marker and therapeutic target for GC and may be one of the markers of TME status transformation.

## Materials and methods

### Raw data collection and processing

RNA-seq and related clinical data in the Gene Expression Omnibus (GEO) database (https://www.ncbi.nlm.nih.gov/geo/ GSE84437) derived from 433 GC patients were downloaded, and the ESTIMATE algorithm was used to assess the extent of TME including stromal and immune cell infiltration, and the stromal scores, immune scores, estimate scores were further estimated. The median values of the immune and stromal scores were classified into two groups of low and high expression, followed by screening of DEGs using the limma package of R software under the criteria of log_2_FC (fold change) in upregulated ≥ 0.5 and downregulated genes ≤ minus 0.5, adjusted p value < 0.05. The heatmap of the relevant DEGs was then plotted using the pheatmap package, and the VennDiagram package was applied to obtain genes associated with both stromal and immune cells to explore the common features of stromal and immune cells. The clinicopathological characteristics of each sample as well as the relationship between overall survival (OS) and the three scoring profiles were then assessed, with visual mapping of results at p < 0.05.

### Functional enrichment analysis and screened for DEG

We conducted functional enrichment analysis for TME-associated DEGs to explore the potential role of DEGs. Biological pathways (BP), cellular components (CC), and molecular functions (MF) as the biological processes in Gene ontology (GO) analysis were employed to explore of DEGs in GC. The Kyoto Encyclopedia of Genes and Genomes (KEGG) shows the results of the pathways. Next, interacting proteins with common biological functions can be visualized through networks. Proteins with similar functions share common topological features in protein–protein interaction (PPI) networks, such as the number of nodes, so to elucidate the potential disease mechanisms in GC and its role in the TME, we mapped PPI networks using the String platform.^1^ The “survival” R package was used to perform univariate Cox regression analysis on DEGs to screen genes associated with GC prognosis, and the top 50 genes at p < 0.05 were plotted. This was followed by a cross-tabulation analysis based on the results of PPI network and univariate Cox regression analysis to filter key genes related to the TME that were associated with both.

### Relationship between CTSK and clinical characteristics and survival of GC patients

The R packages “limma” and “ggpubr” analyzed the clinical characteristics of patients according to their age, gender, T-stage and N-stage, based on the different levels of CTSK gene expression in GC patients. We also investigated the prognostic differences of the two groups with the “survfit” function in the R package to explore the relationship between the CTSK gene and the survival prognosis of GC patients. Next, to further validate the relationship between CTSK expression and the prognosis of tumor patients, we downloaded clinical data with GSE26253 on the GEO platform. GEPIA2 was used to detect the prognosis of expression level in the GC samples [18]. The Kaplan Meier plotter is capable to assess the correlation between the expression of all genes and survival in 30 k + samples from 21 tumor types, it was used to discover and validate whether CTSK is a biomarker in survival prognosis of GC.

### Differential expression of CTSK in pan-cancer

Differential expression of CTSK in various cancers in comparison to adjacent normal tissues was analyzed by downloading the uniformly normalized pan-cancer dataset from the UCSC Xena database (http://xena.ucsc.edu) and further extracting the expression data of ENSG00000143387 (CTSK) gene in each sample. Cancers with fewer than 3 samples in a single specie were eliminated, with 34 cancer species remaining. Each expression value was subsequently log2(x + 1) transformed.

### Relationship between CTSK and TME

To further explore the role of target genes in TME, gene enrichment analysis was performed using the c2.cp.kegg.v7.5.1.symbols.gmt gene set file and GSEA version (4.2.3) to validate the results of the KEGG pathway enrichment analysis and to visually map the pathways showing significant differences. Next, the degree of infiltration of 22 tumor-infiltrating immune cells (TIIC) in GC patients was estimated using the CIBERSORT algorithm and the correlation between each two immune cells in TME was analyzed by Pearson's test. The interrelationship between the expression levels of the CTSK gene and various immune cells was then further explored. Currently, immune checkpoint inhibitors are emerging as a new clinical treatment option for GC. We also examined the correlation between CTSK expression levels and immune checkpoints, and the immune checkpoint genes that met the correlation test for mapping were visualized.

### Screening for relevant sensitivity drugs

To investigate differences of drug in sensitivity between the high and low target gene expression groups, the semi-inhibitory concentrations (IC50) of chemotherapeutic drugs in the two groups was calculated by the pRRophetic package.

### Statistical analysis

R version 4.1.2 was used for all statistical testing and analysis. Non-parametric or parametric method differences were compared using the Wilcoxon test, Kruskal–Wallis test and student-test or one-way ANOVA. Pearson χ^2^ test or Fisher’s exact probability test for comparison of differences in distribution between two groups of categorical variables. Statistical significance was defined as a p-value less than 0.05.

## Results

### Identification and functional analysis of DEGs

**Fig. 1:**
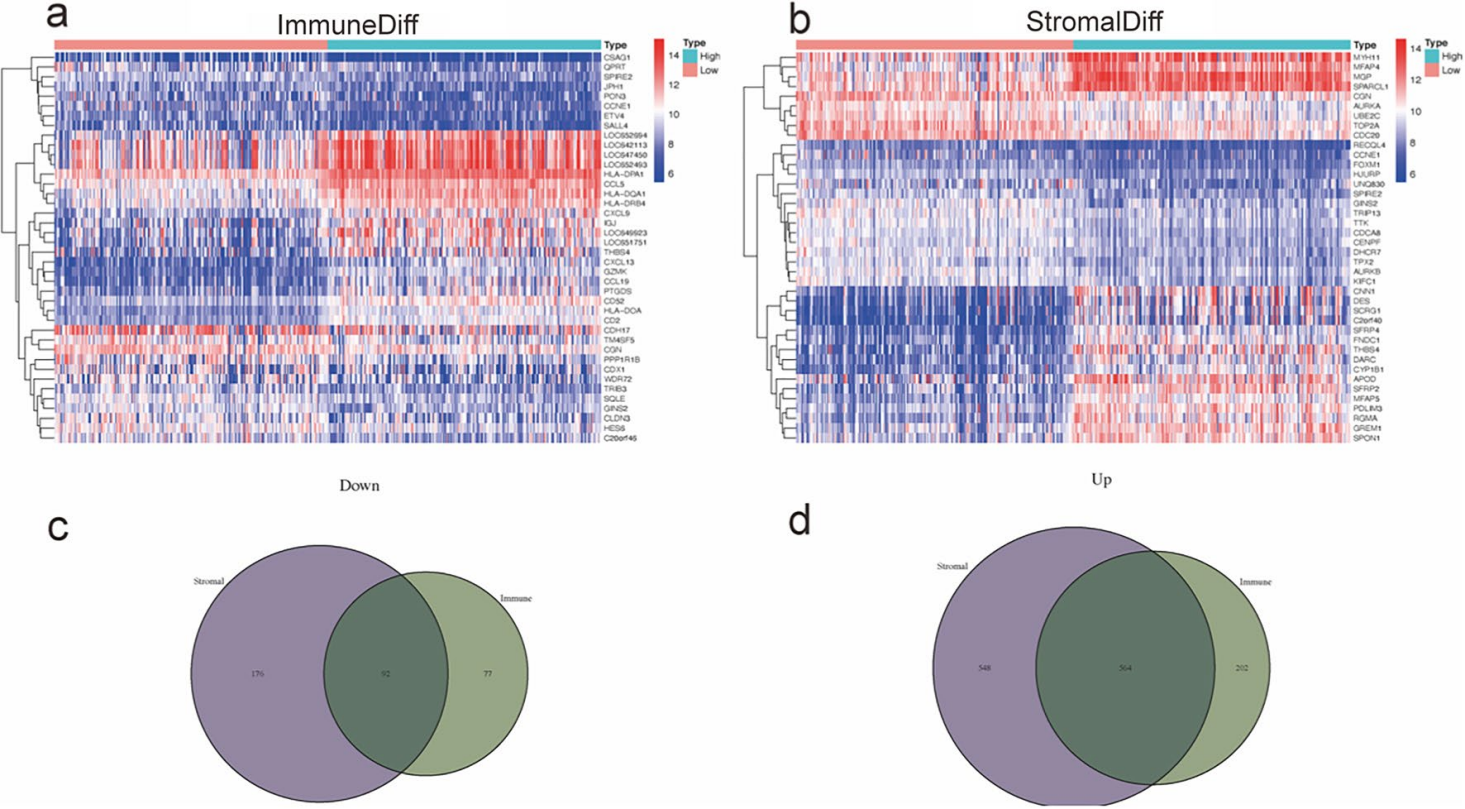
Differential genes in GC patients (**a**). Heatmap of differential genes between high and low scoring groups in immune cells (**b**). Heat map of differential genes between high and low scoring groups in stromal cells (**c**). Venn diagrams of DEGs in low-expressing stromal and immune components (**d**). Venn diagrams of DEG in high expressing stromal and immune components

**Fig. 2:**
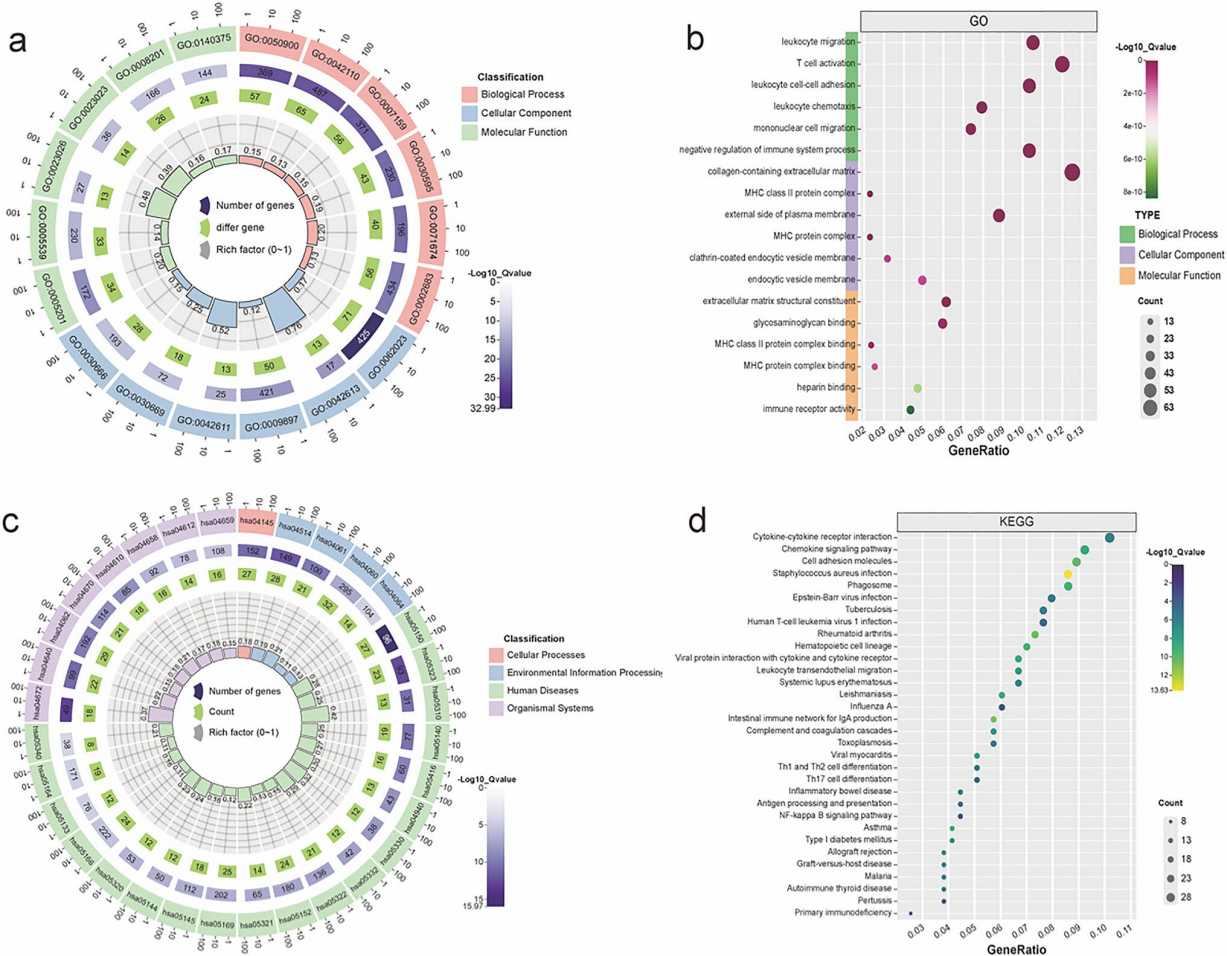
GO and KEGG enrichment analysis of DEGs (**a**). **b** 656 DEGs for molecular functions, cellular components, and biological processes of the top six most significant functions (**c**). **d** 656 DEGs for KEGG enrichment analysis

We analyzed and screened the DEGs based on different stromal scores and immune scores in low immune score samples and high immune score samples, low stromal score samples, high stromal score samples, respectively, and further plotted the heatmap of the top 20 DEGs with low and high expression to determine the gene expression in different components (Fig. 1a, b). We screened 2315 genes in total. A total of 1380 DEGs, including 268 down-regulated genes and 1112 up-regulated genes, were screened according to stromal scores. Similarly, there were 935 DEGs (169 down-regulated genes and 766 up-regulated genes) between high immune scores and low immune scores samples. A total of 656 genes were obtained, which are genes related to the TME (Fig. 1c, d). According to GO analysis (Fig. 2a, b), DEGs are mainly associated with a range of immune responses, such as leukocyte migration and T cell activation. Molecular function shows that DEGs play a potential immunological role in TME by extracellular matrix structural constituent, glycosaminoglycan binding, heparin binding and immune receptor activity. Collagen-containing extracellular matrix, the external side of plasma membrane and the MHC protein complex enhanced in the cellular component. As shown in KEGG analysis, most DEGs were involved in the intestinal immune network for Th17-cell differentiation, cytokine-cytokine receptor interaction, Th17-cell differentiation, Th1 and Th2 cell differentiation and IgA production (Fig. 2c, d). This is a further indication that there is some regulation and control of immune regulation of TME by DEGs.

### The relationship between scoring scores with clinicopathological features and survival

**Fig. 3:**
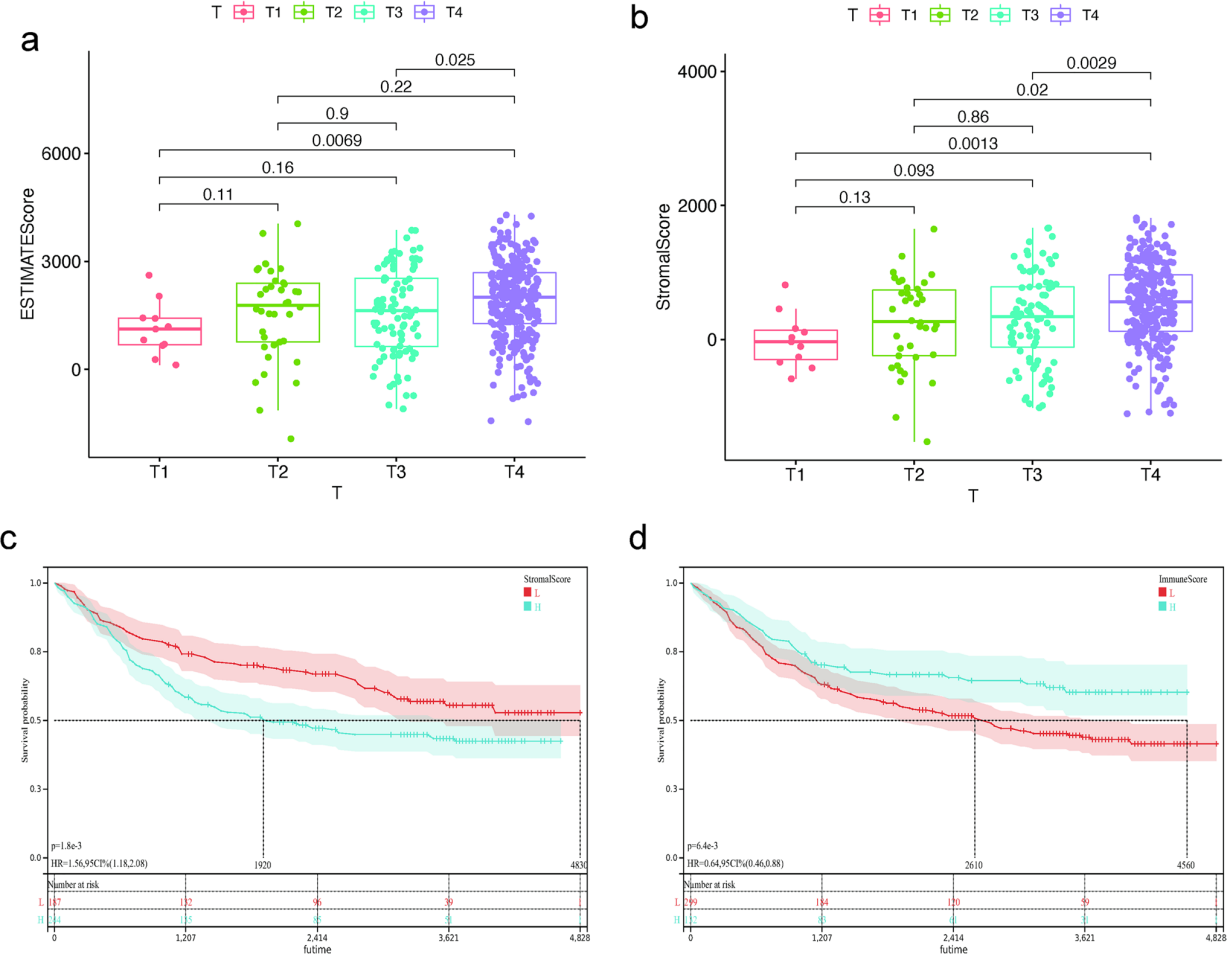
Stromal, immune and composite scores of DEG about GC. (a) Relationship between (b) clinicopathological features and (c-d) prognosis

The corresponding clinical characteristics of 433 patients were analyzed in order to examine the association between the clinical characteristics of patients with GC and Estimate scores, Immune scores, and Stromal scores. The T-stage of the tumor was found to significantly differ from Stromal scores and Estimate scores (p = 0.0013, p = 0.0069, Fig. 3a, b). The Kaplan–Meier method was used to examine the relationship between each score and patient survival in more details. Stromal scores were significantly different and negatively correlated with patient survival [p = 1.8e^−3^, HR = 1.56, 95%CI (1.18, 2.08), Fig. 3c], while Immune scores were significantly positively correlated with patients’ OS [p = 6.4e^−3^, HR = 0.64, 95%CI (0.46, 0.88), Fig. 3d], suggesting that the lower the stromal cells content and the higher the immune cells content in tumor patients, the better prognosis. The Estimate scores and patient survival showed no difference (p = 0.22, Fig. S1). This indicated that the survival prognosis of patients was correlated with the stromal and immune components of TME.

### PPI network and univariate COX regression analysis

**Fig. 4:**
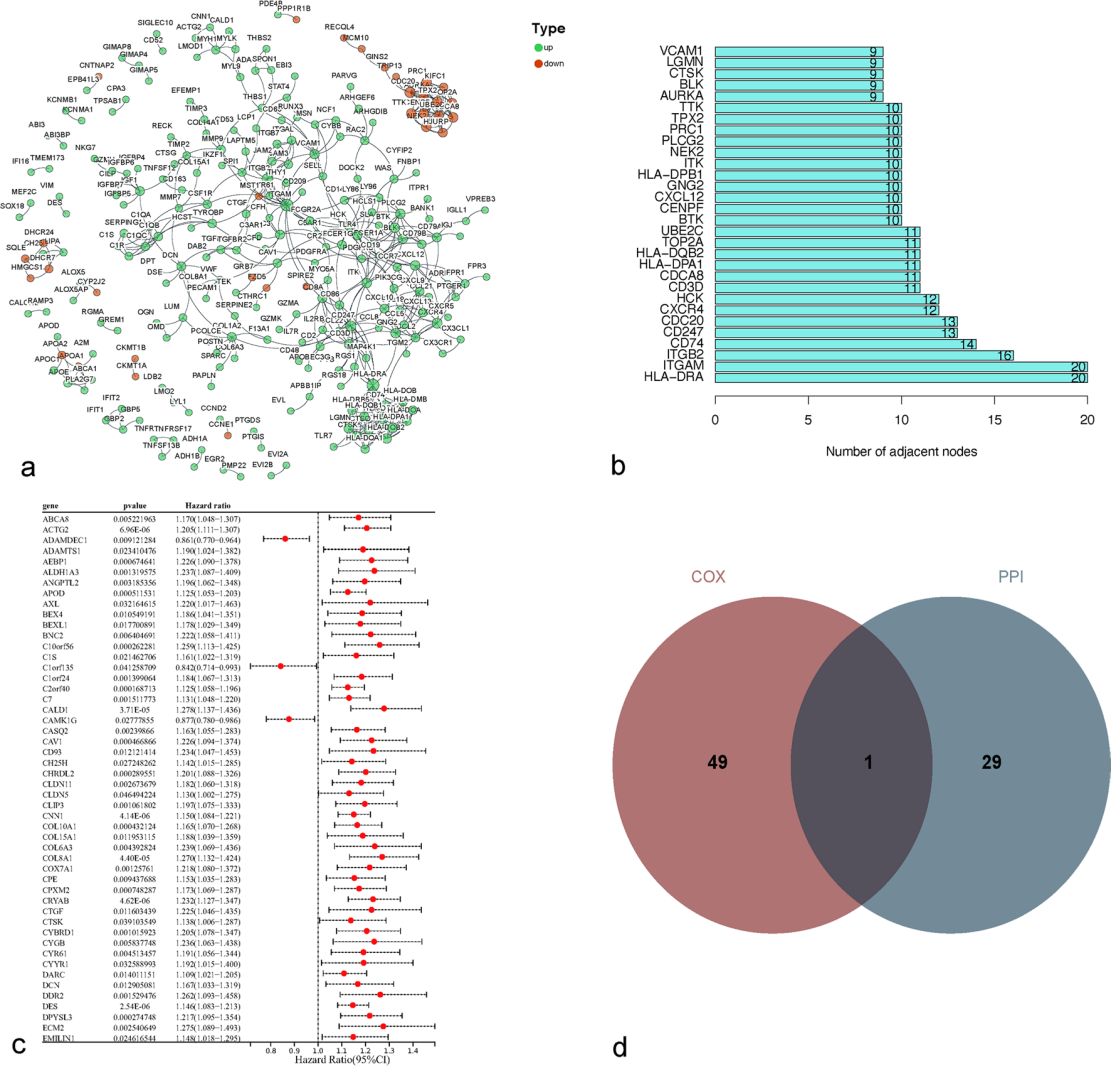
PPI network with univariate COX regression analysis (**a**). PPI network constructed by 656 DEGs, green indicates proteins with up-regulated expression and red indicates proteins with down-regulated expression (**b**). Top 30 genes by the number of adjacent nodes of the PPI network (**c**). Top 50 genes associated with GC prognosis by univariate COX regression analysis (**d**). Cross-tabulation analysis of the PPI network with univariate COX regression analysis yielded CTSK as the only key gene

To further analyze the interactions between DEGs in GC, we constructed a PPI network associated with 656 differential genes using the STRING database, the confidence of the minimum required interaction was 0.900. In the PPI network, a total of 627 nodes, 536 edges, 1.71 average node degree, 0.278 average local clustering coefficient and < 1.0e^−16^ PPI enrichment p value were generated. Then mapped the results of interactions between 263 associated proteins using Cytoscape software (Fig. 4a), and collated the top 30 differential genes in the PPI network in terms of the number of protein-adjacent nodes (Fig. 4b). Univariate COX regression analysis showed that 163 genes were associated with survival risk factors for GC (Fig. 4c). A cross-tabulation analysis of the PPI network results in combination with the results of the univariate COX regression analysis confirmed CTSK as the only overlapping gene (Fig. 4d).

### Pan-cancer expression landscape of CTSK

**Fig. 5:**
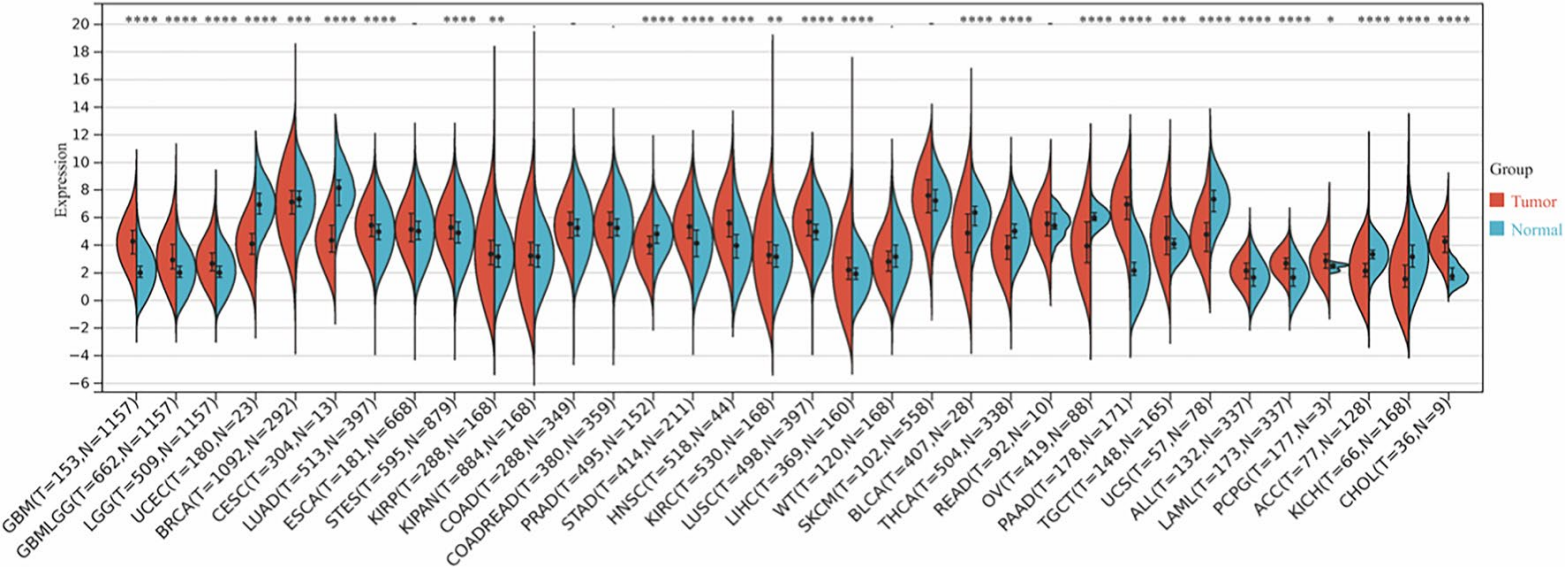
Gene expression of CTSK in 34 cancers Up-regulation of CTSK gene expression: GBM (p = 6.0^e−45^), GBMLGG (p = 1.8e^−65^), LGG (p = 3.6^e−35^), LUAD (p = 7.3e^−8^), STES (p = 1.1^e−11^) KIRP (p = 5.3e^−3^), STAD (p = 2.8e^−20^) HNSC (p = 2.9^e−7^), KIRC (p = 8.1e^−3^), LUSC (p = 4.2e^−15^), LIHC (p = 8.7e^−6^), PAAD (p = 1.2e^−101^), TGCT (p = 3.9e^−4^), ALL (p = 1.4e^−6^), LAML (p = 3.7e^−42^), PCPG (p = 0.04), CHOL (p = 2.4e^−8^), expression of downward adjustment: UCEC (p = 7.5e^−11^), BRCA (p = 1.8e^−4^), CESC (p = 1.5e^−6^), PRAD (p = 1.9e^−15^), BLCA (p = 5.6e^−6^), THCA (p = 7.7e^−46^), OV (p = 5.2e^−60^), UCS (p = 2.6e^−15^), ACC (p = 6.1e^−12^), KICH (p = 1.2e^−7^).*p < 0.05, **p < 0.01, ***p < 0.001, ****p < 0.0001

Based on the analysis in the TCGA GTEx pan-cancer dataset (N = 19,131, G = 60,499), it could be observed that CTSK gene was significantly higher-expressed in 17 cancers such as LUAD, STES, STAD, KIRC, LUSC, LIHC, PAAD, CHOL than their adjacent normal tissues, and that another 10 tumors were observed to be significantly down-regulation, such as UCEC, BRCA, ACC, PRAD, THCA, KICH.CTSK expression was observed to be elevated in tumor tissues in 23 cancer species (Fig. 5). It can be speculated that CTSK may be a potential high-risk gene for cancer, and that its high expression may lead to cancer development.

### Relationship between CTSK expression and clinicopathological features and cancer progression

**Fig. 6:**
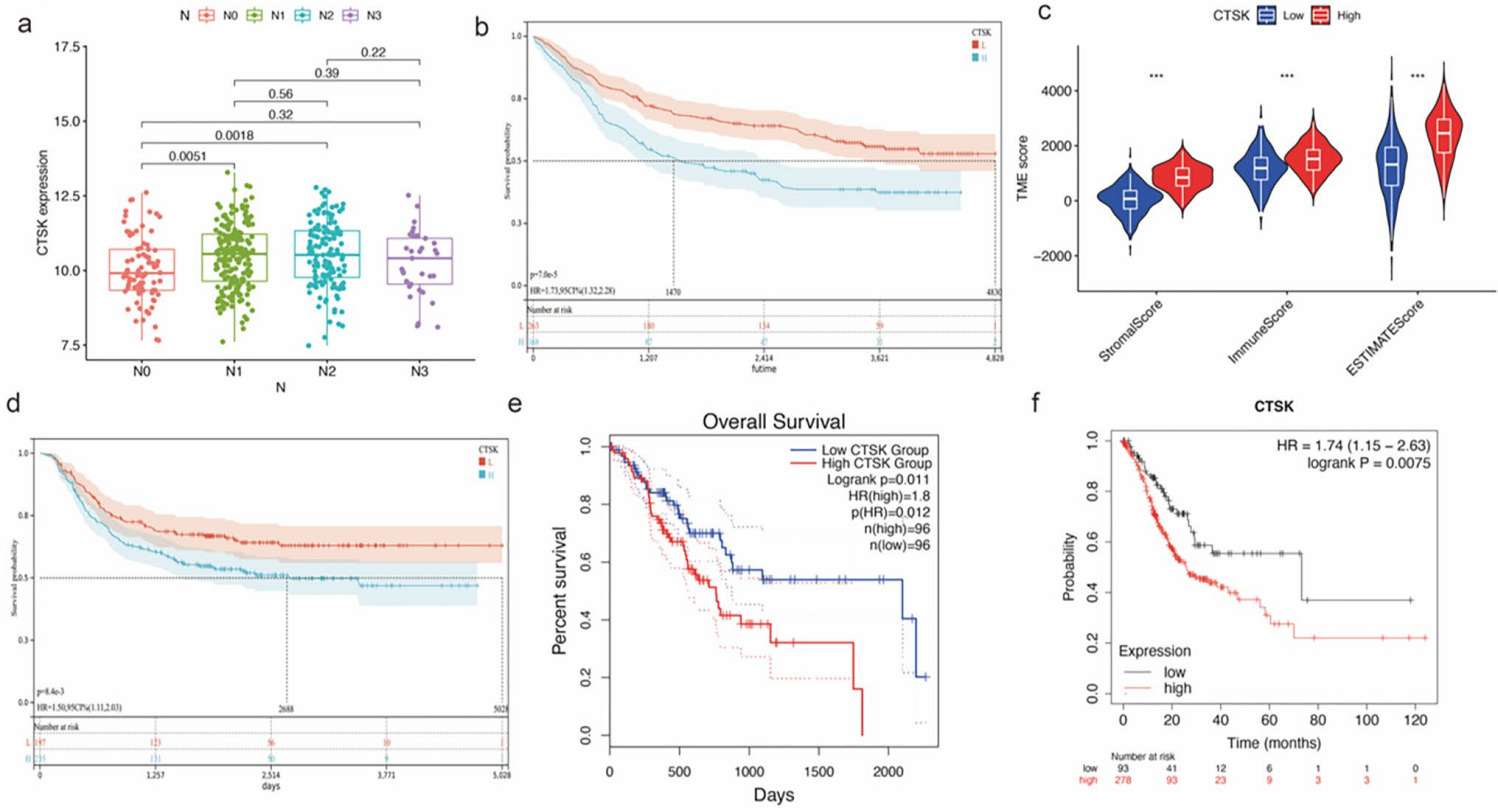
Differences in CTSK gene expression in clinical versus TME (**a**). N-stage (**b**). High CTSK expression leads to lower overall survival (**c**). Differences between high and low expression of CTSK genes in TME between stromal score, immune score and composite score. **d** Validating the RFS relationship of CTSK in dataset GSE26253. **e** Expression levels of the gene CTSK in GEPIA in relation to prognosis. **f** OS analysis of CTSK in GC patients mapped by K-M Plotter

Based on the downloaded clinical data from GC patients, the correlation between patient gender, age, T, N clinicopathological characteristics and survival prognosis with CTSK expression was analyzed. The analysis showed no significant correlation between CTSK expression with gender, age and T-stage of patients (Fig. S2), but there was a significant association with N-stage of lymph node metastasis (p = 0.0018, Fig. 6a). Survival analysis shows that patients with high CTSK expression have 1.73-fold higher risk of death than those with low CTSK expression [p = 7.0e^−5^, HR = 1.73, 95%CI (1.32, 2.28), Fig. 6b]. In addition, high CTSK expression leads to a significantly higher infiltration of stromal cells and immune cells in TME (Fig. 6c). This suggests that CTSK is closely associated with the progression of GC, especially early lymph node metastasis and prognosis. In a further analysis, a survival analysis of the validation set GSE26253 [p = 8.4e^−3^, HR = 1.50, 95%CI (1.11, 2.03), Fig. 6d] and using the GEPIA2 (p = 0.011, HR = 1.8, Fig. 6e) and K-M plotter [p = 0.0075, HR = 1.74, 95%CI (1.15, 2.63), Fig. 6f] platform revealed that OS as well as recurrence free survival (RFS) was significantly lower for patients in the CTSK high expression group than in the CTSK low expression group.

### The relationship between CTSK and GC TME and tumor immune response

**Fig. 7:**
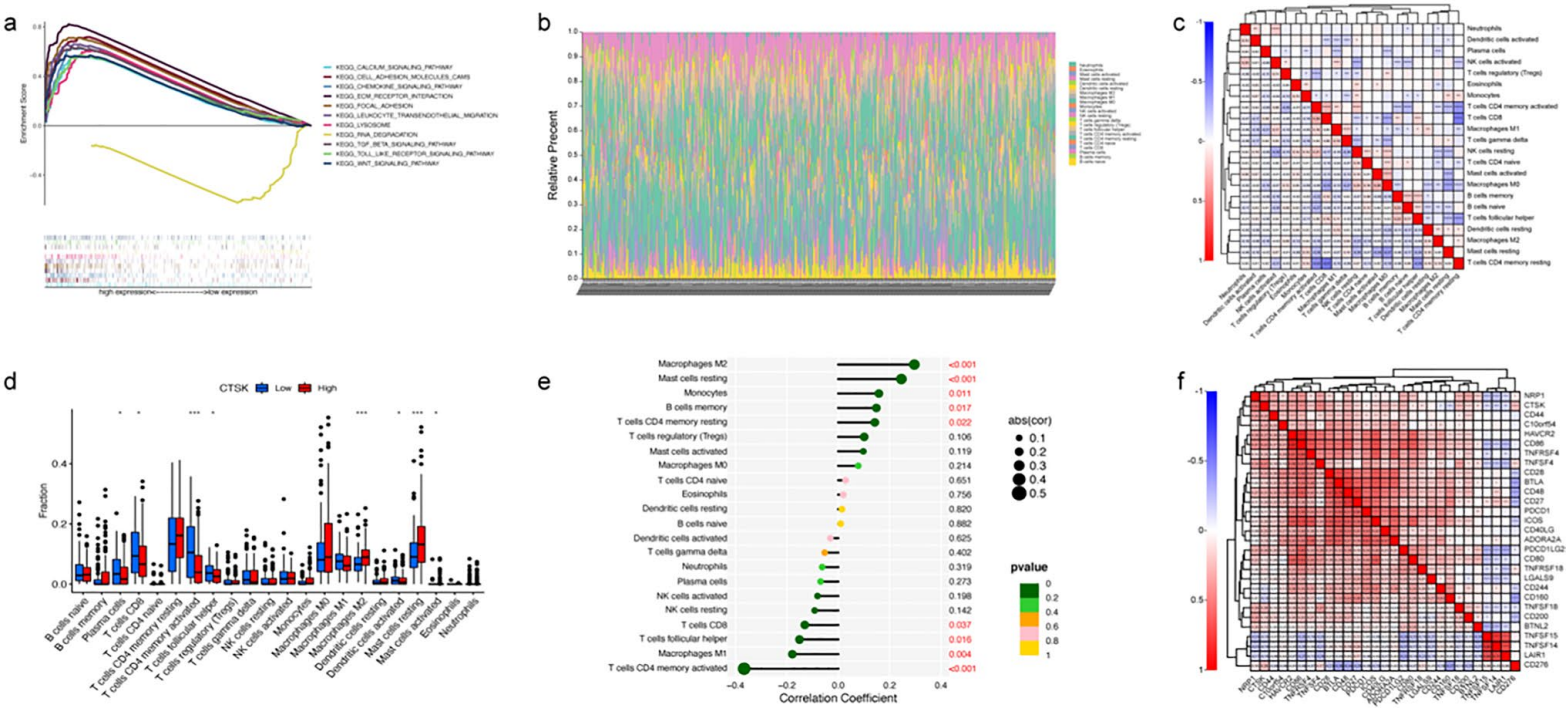
Potential pathways of CTSK and immune response relationships. **a** GSEA enrichment results of CTSK. **b** Distribution of 22 types of immune cell infiltration in each GC patient. **c** Interrelationship between 22 types of immune cells. **d** box plot of the proportional difference in the distribution between TIIC and the two groups with high and low CTSK expression. **e** Correlation between CTSK expression and 22 TIICs. **f** Correlation between CTSK and immune checkpoint genes. (*p < 0.05, **p < 0.01, ***p < 0.001)

CTSK genes were found to be involved in the regulation of numerous GC immunological responses and signaling pathways by GSEA enrichment analysis. Among them, the chemokine signaling pathway, WNT signaling pathway, TGF-βsignaling pathway, etc. were significantly correlated with high CTSK expression, while RNA degradation was negatively correlated with CTSK expression (Fig. 7a). After analyzing the infiltration of 22 TIICs in GC tumor tissue and the correlation between immune cells, we found that the interaction between T lymphocytes and Tumor-Associated Macrophages (TAM) was more prominent (Fig. 7b, c). When the distribution of TIIC in GC tumors samples with low or high expression of CTSK was further analyzed, it was discovered a considerably larger proportion of TAM in the high CTSK expression group than the low expression group (Fig. 7d), and we found that CTSK expression was closely associated with nine immune cell types (Fig. 7e). Of these, macrophage M2 cells, mast resting cells, monocytes, CD4 memory resting T cells, B memory cells had positive correlations, and four were negatively correlated, including CD8 T cells, follicular helper T cells, memory activated CD4 T cells, and macrophage M1 cells. The correlation between 47 common immune checkpoint genes and CTSK expression are shown in (Fig. 7f). From the above results, it can be concluded that CTSK played an important role in the TME and immune response of GC that may become a new immunotherapeutic target for tumor treatment.

### Analysis of CTSK expression levels and drug sensitivity

**Fig. 8:**
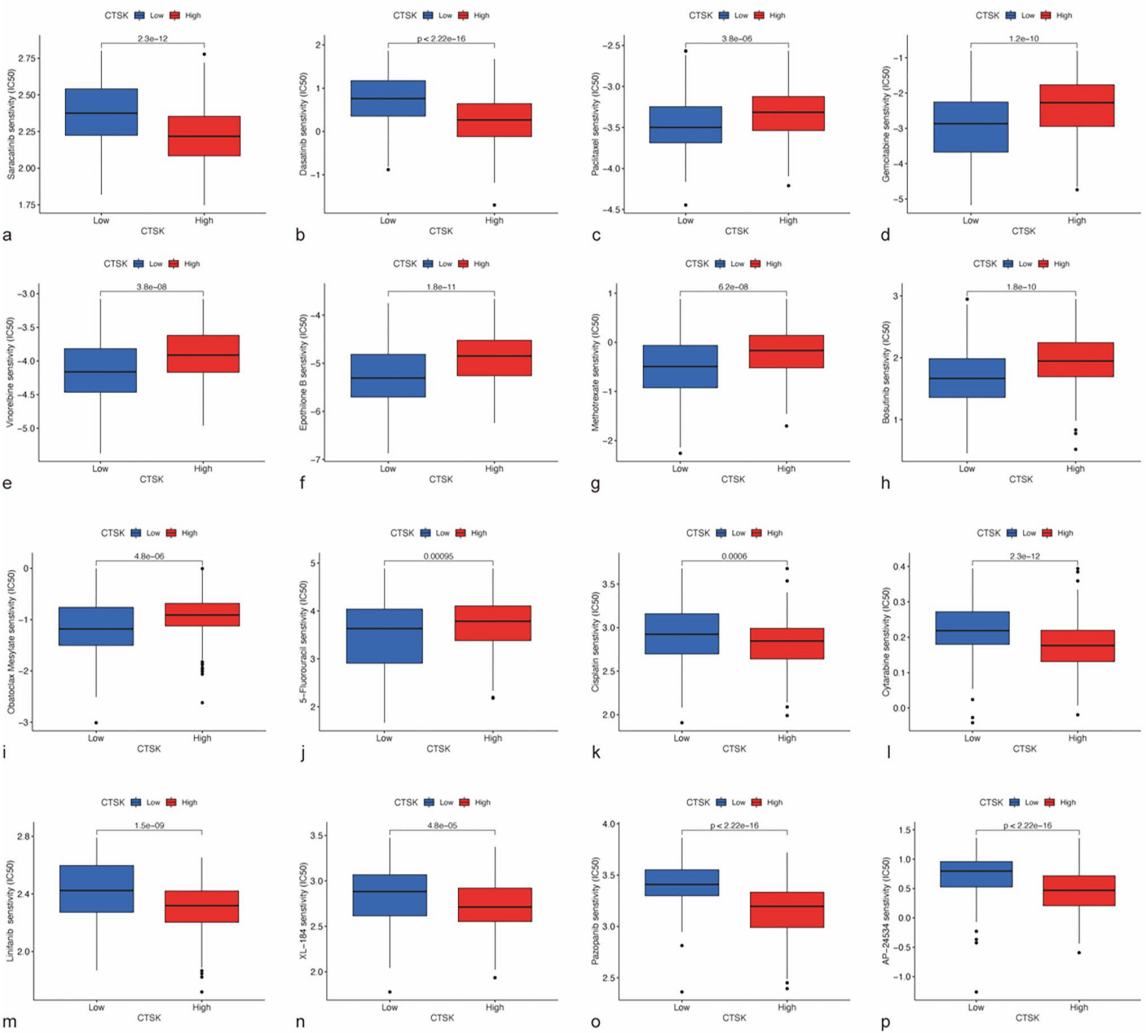
Analysis of the sensitivity of high versus low CTSK expression groups to different drugs

To determine whether the CTSK gene could be used as a biomarker for responding to the efficacy of drug therapy in GC patients, the IC50 values of various antitumor drugs were estimated. From the results, we observed that patients with lower levels of CTSK expression were more sensitive to treatment with Bosutinib, Methotrexate, Epothilone, Paclitaxel, 5-Fluorouracil, Obatoclax Mesylate and cell cycle-specific drugs Vinorelbine that effectively block cell division, while patients with high levels of CTSK expression responded more positively to Cisplatin, Cytarabine, pazopanib and multi-target inhibitors such as Linifanib, Cabozantinib (XL-184), Dasatinib, Saractinib, Ponatinib (AP-24534). Overall, there was a correlation between CTSK expression and sensitivity to drug treatment in GC (Fig. 8).

## Discussion

The development of tumor is closely linked to their own heterogeneity and TME drivers, studies on the two for identifying sensitive and specific prognostic biomarkers could improve early diagnosis and significantly extend cancer patients’ survival time. In the present study, we used gene expression data from the GEO database and identified CTSK as a gene associated with survival prognosis, TMN staging and possibly played an important role in the TME component in GC patients.

Cathepsin-K is usually studied as a functional molecule related to osteoclasts, belonging to the cathepsin L-like cluster of the C1A family, elevated CTSK activity is strongly associated with the development and recurrence of metastasis in many cancers [19]. CTSK has been shown to promote invasion and metastasis of breast cancer through direct degradation of the extracellular matrix (ECM) and promotion of angiogenesis or enhancing paracrine interactions [20, 21]. High expression of CTSK enhances the invasiveness of prostate cancer cells and provides anchor points for cancer cells, playing a key role in lymph node metastasis and bone metastasis in prostate cancer [22–24]. CTSK expression could be detected in lung tissues such as bronchial and alveolar epithelium and alveolar macrophages [25], however, CTSK was significantly elevated in TAM in advanced NSCLC [26], which may be related to the fact that CTSK could promote the proliferation of A549 cells by directly activating the mTOR signaling pathway, thereby promoting the progression of NSCLC [27]. In colorectal cancer (CRC), CTSK can act as a mediator of intestinal microbiota imbalance leading to metastasis of CRC [28]. Another CTSK expression upregulation is GC in which coronin 3 promotes metastasis through upregulation of MMP-9 and CTSK expression and shows a strong link with GC lymph node metastasis and unfavorable prognosis [29]. This is consistent with our results, in which patients with high CTSK expression in this study had a significantly worse prognosis than those with low expression, and in the validation set it was also found that RFS was lower in patients with high expression. Furthermore, the promotion of ECM breakdown by stromal fibroblasts that are CTSK-positive is crucial for the development of tumors as it could promote invasive squamous cell carcinoma (SCC) growth and invasion into surrounding tissues and blood vessels [30]. Therefore, CTSK may be a potential biomarker for early diagnosis of GC.

TIICs in TME play a key role in cancer, and different subtypes of immune cells may drive tumor progression, metastasis and resistance to treatment [31]. This study applied the CIBERSORT algorithm to examine the landscape of immune cells that had infiltrated tumors and found a positive link of immune cell infiltration with expression of CTSK. Such as M2 macrophages cells, resting mast cells, monocytes cells, and negatively related to M1 macrophages cells, suggesting that the CTSK level was closely related to TAM.TAM as an important part of TME is an important regulators of tumor-associated inflammation [32]. It has been found that mast cells promote GC development through the release of angiogenic factors and lymphangiogenic factors and are associated with angiogenesis in tumor tissue, lymph node metastases and patient survival [33]. Monocytes can influence TME through a variety of mechanisms while further inducing immune tolerance, tumor angiogenesis and metastasis of tumor cells [34]. Similarly, CTSK can promote CRC progression by promoting M2 polarization of TAM through a TLR4-mTOR-dependent pathway, while M2 macrophages can secrete chemokines such as IL7 and EGFR to activate the NFκB pathway to further promote tumor tissue invasion and metastasis [28, 35]. TAM in TME can be polarized into both M1 (anti-tumor) and M2 (pro-tumor) phenotypes, while CTSK expression level was positively correlated with M2 macrophages and can drive M2 polarization of TAM. Taken together we can infer that the evidence of interaction between tumor-infiltrating immune cells and CTSK in TME supported the potential of CTSK as a novel GC immunotherapeutic target.

Next, the relationship between CTSK expression and immunological checkpoint molecules was studied. The data showed that CTSK expression and NRP1 in GC significantly correlated positively, suggesting that we could target the relevant immune checkpoint genes to improve the immune efficacy of GC. NRP1 is a neuronal and endothelial cell receptor, which can interact with ibronectin-1 (FN1) to promote GC progression by remodeling the ECM [36]. We hypothesized that CTSK was involved in angiogenesis and stromal remodeling in tumor tissue [37], and similarly, NRP1 induced epithelial-mesenchymal transition (EMT) by promoting GC metastasis via activating PI3K/Akt signaling pathway [38]. On the other hand, the level of infiltration of Macrophages M2 cells was positively correlated with CTSK expression in TIICs, while NRP1 expression can significantly increase the infiltration of immunosuppressive cells, such as Tregs and Macrophages M2 [39]. The same role of NRP1 and CTSK in TIIC further predicts its potential therapeutic role. Several studies have demonstrated that small molecule inhibitors such as small interfering RNAs [40], peptides [41], and monoclonal antibodies [42] can exert targeted therapeutic effects through inhibiting the oncogenic activity of NRP1. Anti-NRP1-mAb treatment inhibits the phosphorylation of Akt in GC cells and down-regulates VEGF expression in vivo to inhibit GC recurrence and metastasis [43]. Furthermore, NRP1, a novel immune checkpoint, selectively affects the production of T cell memory precursors during antitumor immune response in a cell-intrinsic manner, suggesting that NPR1 combined with immune checkpoint blockade therapy can effectively clear tumors, enabling the combined blockade of NRP1 and PD1 and contribute to more positive clinical outcome for tumor patients [44]. In this study, we found that the expression of CTSK in GC shared a common mechanism of action with NRP1, and we hypothesized that the interaction between CTSK, NRP1 and PD-1/PD-L1 in GC had far-reaching clinical implications for the immune-targeted therapy of GC.

This study also had certain limitations. Firstly, the experimental data lacked more detailed clinical information, pointing to a gap between CTSK and more detailed clinicopathological features of tumor patients. Secondly, there was a lack of corresponding experimental evidence to demonstrate that CTSK functioned in angiogenesis and ECM remodeling in tumor development. Finally, CTSK combined with NRP1 and PD-1/PD-L1 immune-targeted therapy should be further validated in clinical practice.

## Conclusion

Overall, we used the GC patients’ data in the GEO public database to show that CTSK may be a potential prognostic biological marker for GC through analyzing the differential genes between TME stromal cells and immune cells. The current findings supported a critical role of CTSK in tumor development and explored the relationship between CTSK and TME of GC, immune checkpoints and tumor-infiltrating immune cells. The results confirm that CTSK is a promising predictive target related to the TME of gastric cancer, and that its expression not only predicts patient prognosis but also provides new strategies for immunotherapy of tumor.

### Supplementary Information

Additional file1 (DOCX 7211 KB)

## Data Availability

The datasets generated during and/or analysed during the current study are available from the corresponding author on reasonable request. All data generated or analysed during this study are included in this published article and its supplementary materials files. Further inquiries can be directed to the corresponding author.

## Footnotes

1 http://string-db.org/..

## References

[1] Smyth EC (2020). Gastric cancer. Lancet.

[2] Torre LA (2016). Global cancer incidence and mortality rates and trends—an update. Cancer Epidemiol Biomarkers Prev.

[3] Xia C (2022). Cancer statistics in China and United States, 2022: profiles, trends, and determinants. Chin Med J.

[4] Green PH (1988). Increasing incidence and excellent survival of patients with early gastric cancer: experience in a United States medical center. Am J Med.

[5] Li K (2021). Advances in clinical immunotherapy for gastric cancer. Biochim Biophys Acta Rev Cancer.

[6] Glimelius B (1997). Randomized comparison between chemotherapy plus best supportive care with best supportive care in advanced gastric cancer. Ann Oncol.

[7] Zeng D (2018). Gene expression profiles for a prognostic immunoscore in gastric cancer. Br J Surg.

[8] Jiang Y (2018). ImmunoScore signature: a prognostic and predictive tool in gastric cancer. Ann Surg.

[9] Pitt JM (2016). Targeting the tumor microenvironment: removing obstruction to anticancer immune responses and immunotherapy. Ann Oncol.

[10] Zhang B (2020). m(6)A regulator-mediated methylation modification patterns and tumor microenvironment infiltration characterization in gastric cancer. Mol Cancer.

[11] Hanahan D, Coussens LM (2012). Accessories to the crime: functions of cells recruited to the tumor microenvironment. Cancer Cell.

[12] Zeng D (2021). Tumor microenvironment evaluation promotes precise checkpoint immunotherapy of advanced gastric cancer. J Immunother Cancer.

[13] Ishimoto T (2014). Interaction between gastric cancer stem cells and the tumor microenvironment. J Gastroenterol.

[14] Galon J (2006). Type, density, and location of immune cells within human colorectal tumors predict clinical outcome. Science.

[15] Song C (2019). Syringeable immunotherapeutic nanogel reshapes tumor microenvironment and prevents tumor metastasis and recurrence. Nat Commun.

[16] Li W (2021). Infiltrating immune cells in gastric cancer: a novel predicting model for prognosis. J Cancer.

[17] Gu Y (2021). Role of CXCR4 as a prognostic biomarker associated with the tumor immune microenvironment in gastric cancer. Front Cell Dev Biol.

[18] Tang Z (2017). GEPIA: a web server for cancer and normal gene expression profiling and interactive analyses. Nucleic Acids Res.

[19] Novinec M, Lenarčič B (2013). Cathepsin K: a unique collagenolytic cysteine peptidase. Biol Chem.

[20] Söderström M (2001). Cysteine proteinases in chondrosarcomas. Matrix Biol.

[21] Martignoni G (2011). Differential expression of cathepsin K in neoplasms harboring TFE3 gene fusions. Mod Pathol.

[22] Rao Q (2013). Cathepsin K expression in a wide spectrum of perivascular epithelioid cell neoplasms (PEComas): a clinicopathological study emphasizing extrarenal PEComas. Histopathology.

[23] Argani P (2010). A distinctive subset of PEComas harbors TFE3 gene fusions. Am J Surg Pathol.

[24] Balic M (2006). Most early disseminated cancer cells detected in bone marrow of breast cancer patients have a putative breast cancer stem cell phenotype. Clin Cancer Res.

[25] Caliò A (2018). Cathepsin K expression in clear cell “sugar” tumor (PEComa) of the lung. Virchows Arch.

[26] Wang R (2011). Tumor-associated macrophages provide a suitable microenvironment for non-small lung cancer invasion and progression. Lung Cancer.

[27] Yang H (2020). The potential role of cathepsin K in non-small cell lung cancer. Molecules.

[28] Li R (2019). Gut microbiota-stimulated cathepsin K secretion mediates TLR4-dependent M2 macrophage polarization and promotes tumor metastasis in colorectal cancer. Cell Death Differ.

[29] Ren G (2012). Coronin 3 promotes gastric cancer metastasis via the up-regulation of MMP-9 and cathepsin K. Mol Cancer.

[30] Yan X (2011). Stromal expression of cathepsin K in squamous cell carcinoma. J Eur Acad Dermatol Venereol.

[31] Galli F (2020). Relevance of immune cell and tumor microenvironment imaging in the new era of immunotherapy. J Exp Clin Cancer Res.

[32] Mantovani A (2017). Tumour-associated macrophages as treatment targets in oncology. Nat Rev Clin Oncol.

[33] Sammarco G (2019). Mast cells, angiogenesis and lymphangiogenesis in human gastric cancer. Int J Mol Sci.

[34] Ugel S (2021). Monocytes in the tumor microenvironment. Annu Rev Pathol.

[35] Popēna I (2018). Effect of colorectal cancer-derived extracellular vesicles on the immunophenotype and cytokine secretion profile of monocytes and macrophages. Cell Commun Signal.

[36] Wu C (2020). Neuropilin-1 interacts with fibronectin-1 to promote epithelial-mesenchymal transition progress in gastric cancer. Onco Targets Ther.

[37] Krieg AM, Lipford GB (2008). Immunology: the toll of cathepsin K deficiency. Science.

[38] Jin Q (2021). Neuropilin-1 predicts poor prognosis and promotes tumor metastasis through epithelial-mesenchymal transition in gastric cancer. J Cancer.

[39] Kang JY, Gil M, Kim KE (2020). Neuropilin1 expression acts as a prognostic marker in stomach adenocarcinoma by predicting the infiltration of Treg cells and M2 macrophages. J Clin Med.

[40] Bergé M (2010). Small interfering RNAs induce target-independent inhibition of tumor growth and vasculature remodeling in a mouse model of hepatocellular carcinoma. Am J Pathol.

[41] Barr MP (2005). A peptide corresponding to the neuropilin-1-binding site on VEGF(165) induces apoptosis of neuropilin-1-expressing breast tumour cells. Br J Cancer.

[42] Grandclement C, Borg C (2011). Neuropilins: a new target for cancer therapy. Cancers.

[43] Ding Y (2018). Anti-neuropilin-1 monoclonal antibody suppresses the migration and invasion of human gastric cancer cells via Akt dephosphorylation. Exp Ther Med.

[44] Liu C (2020). Neuropilin-1 is a T cell memory checkpoint limiting long-term antitumor immunity. Nat Immunol.

