# Supplementary material for: Prediction and therapeutic targeting of the tumor microenvironment-associated gene CTSK in gastric cancer
Source: Discov Oncol. 2023 Nov 6;14:200. doi: 10.1007/s12672-023-00821-0 (PMC10628060; doi:10.1007/s12672-023-00821-0)
Supplement: Supplementary file 1 — Additional file1 (DOCX 7211 KB) [file 12672_2023_821_MOESM1_ESM.docx]

**Supplementary Material**

**
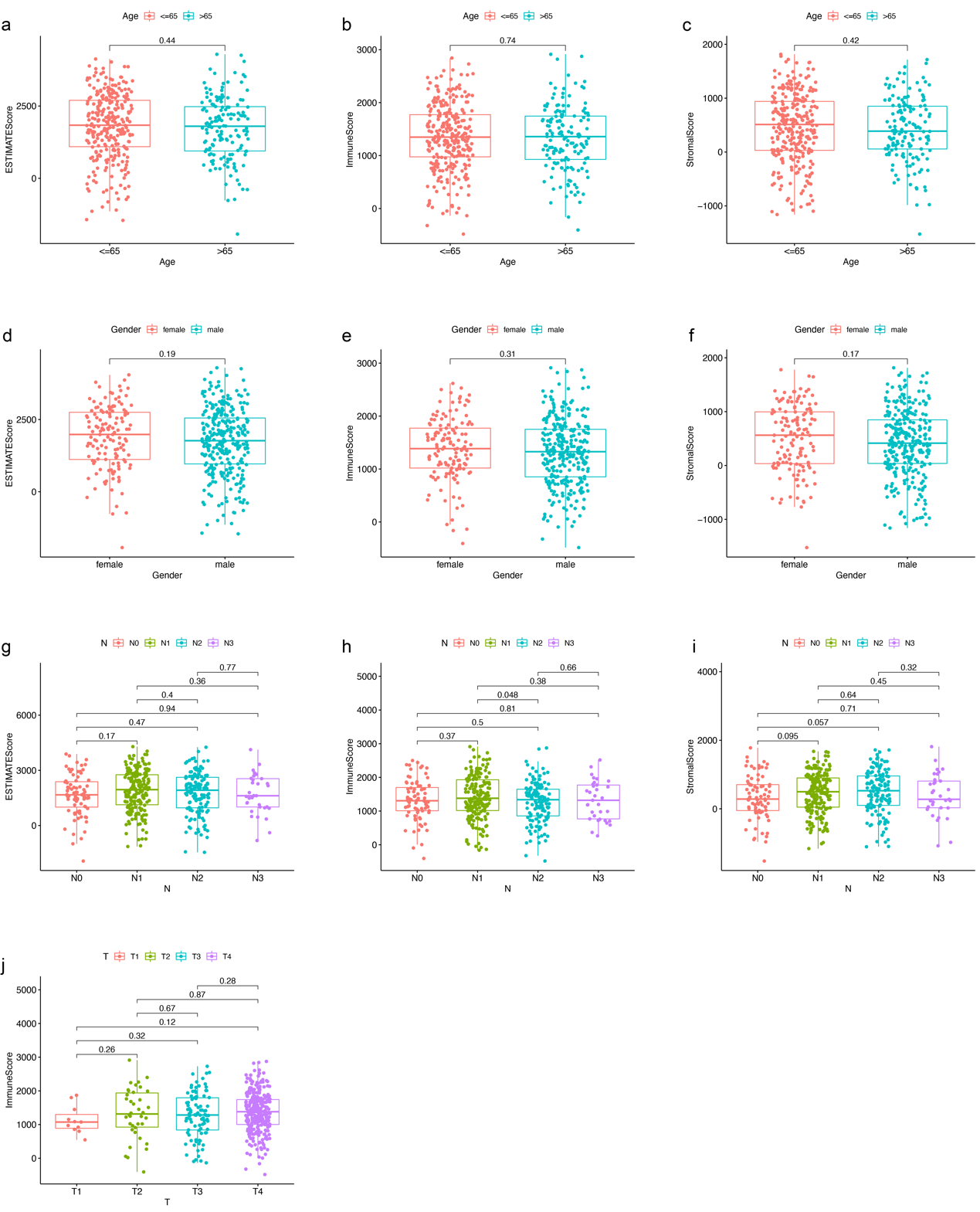
**

FigS1. Relationship between clinical features and pathology with immune, stromal and estimate scores (a-j).

**
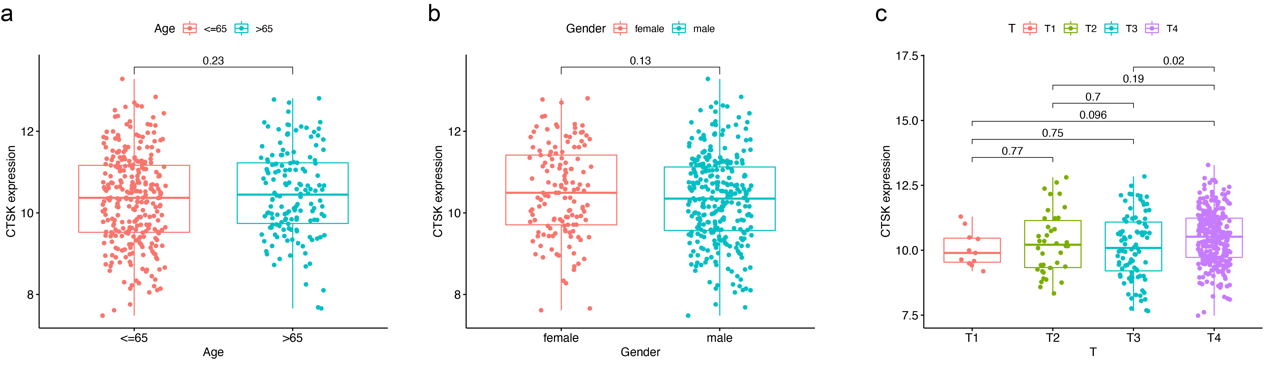
**

FigS2. Relationship between clinical features and pathology with CTSK expression (a-c).
